# Analysis of Microbial Communities in Membrane Biofilm Reactors Using a High-Density Microarray

**DOI:** 10.3390/membranes13030324

**Published:** 2023-03-11

**Authors:** Shilong Li, Liang Duan, Yang Zhao, Fu Gao, Slawomir W. Hermanowicz

**Affiliations:** 1 State Key Laboratory of Environmental Criteria and Risk Assessment, Chinese Research Academy of Environmental Sciences, Beijing 100012, China; 2 Department of Civil and Environmental Engineering, University of California, Berkeley, CA 94720-1710, USA; 3 Technical Centre for Soil, Agriculture and Rural Ecology and Environment, Ministry of Ecology and Environment, Beijing 100012, China

**Keywords:** high-density microarray, microbial community, membrane biofilm reactor, operation time, nitrate removal

## Abstract

Membrane biofilm reactors (MBfRs) have attracted more and more attention in the field of wastewater treatment due to their advantages of high mass transfer efficiency and low-carbon emissions. There are many factors affecting their nitrogen removal abilities, such as operation time, electron donor types, and operation modes. The operation time is directly related to the growth status of microorganisms, so it is very important to understand the effect of different operation times on microbial composition and community succession. In this study, two parallel H_2_-based MBfRs were operated, and differences in microbial composition, community succession, and NO_3_^−^-N removal efficiency were investigated on the 30th day and the 60th day of operation. The nitrogen removal efficiency of MBfRs with an operation time of 60 days was higher than that of MBfRs with an operation time of 30 days. *Proteobacteria* was the dominant phylum in both MBfRs; however, the composition of the microbial community was quite different. At the class level, the community composition of *Proteobacteria* was similar between the two MBfRs. *Alphaproteobacteria* was the dominant class in MBfR, and *Betaproteobacteria* and *Gammaproteobacteria* were also in high proportion. Combined with the analysis of microbial relative abundance and concentration, the similarity of microbial distribution in the MBfRs was very low on day 30 and day 60, and the phylogenetic relationships of the top 50 dominant universal bacteria and *Proteobacteria* were different. Although the microbial concentration decreased with the extension of the operation time, the microbial abundance and diversity of specific functional microorganisms increased further. Therefore, the operation time had a significant effect on microbial composition and community succession.

## 1. Introduction

With increasing water shortages, there is an urgent need to study green wastewater treatment technologies. The combination of membrane technologies and biological treatment technologies provides new ideas for wastewater treatment, and there are more and more green treatment technologies that combine the two [1,2,3,4,5]. The membrane biofilm reactor (MBfR) is a new technology for wastewater treatment. It can be used to remove pollutants such as organic matter, nitrate, and heavy metals [6,7]. It has received increasing attention in recent years. MBfR uses microporous hollow fiber membranes to transfer hydrogen gas to microorganisms on the biofilm and participates in the pollutants removal process as the electron donor [8]. Different from conventional aeration, MBfR has a high electron utilization efficiency due to the non-bubble aeration [9]. It can also use methane and carbon monoxide as electron acceptors, which is an environmentally friendly low-carbon technology. MBfR has a unique microbial community structure and the microbial community composition is directly related to its performance. The microbial communities in bioreactors are affected by many factors, such as operation time, operation modes, and sludge retention times [10,11]. The growth status of microorganisms in the bioreactors is closely related to their operation time [12,13]; therefore, studying the effect of operation time on microbial composition and community succession is of great significance to reveal the mechanism of MBfR action.

Taxonomic acid-based analysis methods are commonly used to study the status of microbial communities, which, to a great extent, are limited by the speed of sequence analysis. The high-throughput technology of microarrays can overcome this shortcoming and simultaneously realize the detection, identification, characterization, and quantification of microorganisms in the natural environment [14]. High-density microarrays can classify and identify large microbial communities without the need for subsequent DNA isolation and sequencing. Due to numerous advantages, this technique is already being used in the studies of environmental microbes [15,16,17].

In this study, we operated two parallel H_2_-based MBfRs. To provide microbial information support for the continuous development of MBfRs, we used high-density microarray technology, PhyloChips including 297,851 probes targeted to 8935 clusters in 16S rRNA gene sequences, to determine microbial information in the bioreactors. Combined with the nitrogen removal efficiency of MBfRs, we analyzed the microbial composition and community succession of MBfRs with different operation times (30 days and 60 days).

## 2. Materials and Methods

### 2.1. Membrane Biofilm Reactor (MBfR) Operation

**Figure 1:**
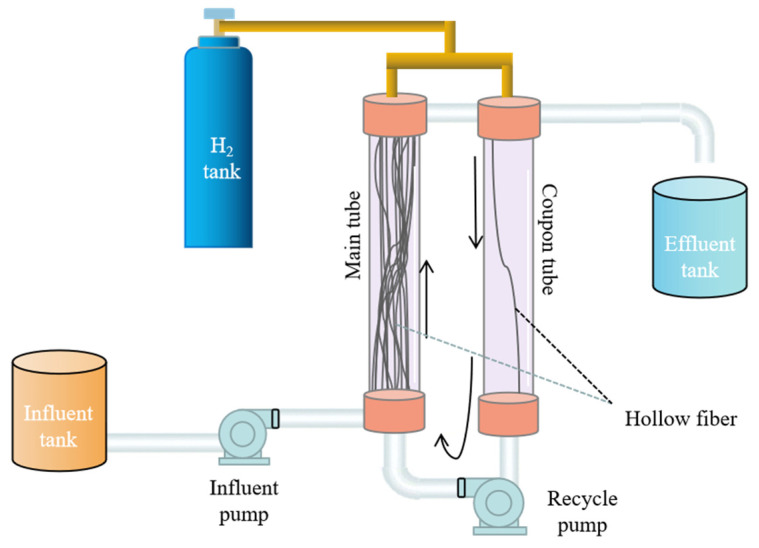
The composition and structure of the MBfR.

The MBfR used in this study consisted of two membrane modules connected to a circulating pathway (Figure 1). A single peristaltic pump ensured that the water circulated through the reactor and the system was thoroughly mixed. The main membrane tube assembly consisted of 32 hollow fiber membranes and the coupon tube assembly consisted of 1 hollow fiber membrane. The fiber membrane in the coupon tube was used to collect microbial samples. One end of the membrane was closed to ensure the diffusion of hydrogen gas from the membrane pores into the wastewater. The inner diameter of the main and the coupon tube was 6 cm, the outer diameter of the hollow fiber membrane made of polyvinyl chloride (PVC) was 0.15 cm, the inner diameter was 0.085 cm, the pore size was 0.01 μm, and the total surface area was 217.70 cm^2^.

### 2.2. Analytical Methods for Water Quality Parameters

The influent and effluent water samples were collected, and the nitrogen removal performance of the MBfRs was monitored by analyzing the influent and effluent water. Total nitrogen and nitrate nitrogen were measured using the Hach Methods 8039 and 8008 according to the manufacturer’s instructions.

### 2.3. High-Density Microarray

In this study, a G2 microarray chip designed by Lawrence Berkeley National Laboratory was used to identify bacterial species. The microarray consists of 506,944 probes. Of these features, 297,851 probes targeted 8935 clusters of 16S rRNA gene sequences, and the rest were used for image orientation and normalization controls. The sequences were clustered according to the 97% similarity degree, and each class formed was called an operational taxon (OTU). The average number of probes per OTU was 24. 

The oligonucleotides selected for this microarray chip were synthesized directly by Affymetrix Inc. (Santa Clara, CA, USA) onto a square glass surface (1.28 × 1.28 cm) via a photolithographic method. The density of oligonucleotides was about 10,000 molecules/um^2^. The features of the probe were arranged in a square grid (712 rows and columns). Each unique probe sequence (feature) on the array occupies a square with a side length of 18 mm and a copy number of approximately 3.2 × 10^6^.

Analysis procedures of the microarray included DNA fragmentation, terminal labeling, prehybridization, hybridization, staining, washing, and scanning. Individual signal values and intensities were available after PhyloChip scanning was complete. 

### 2.4. Phylogenetic Analysis

MEGA11 software (https://www.megasoftware.net/dload_mac_beta, accessed on 20 February 2023) was used to analyze the phylogenetic tree of all detected microbial gene sequences. First, all gene sequences were aligned by ClustalW, followed by a phylogenetic tree built using a neighbor-joining method with 1000 fast bootstrapping replicates. Finally, phylogenetic tree files in the nwk format were visualized using the online tool iTOL (http://itol.embl.de, accessed on 20 February 2023). The specific steps to beautify the phylogenetic tree are as follows: first, adjust the phylogenetic tree to a circular mode, then create the same color range for the same species of microbes, and finally, label the species.

## 3. Results and Discussion

### 3.1. The Process Performance

**Figure 2:**
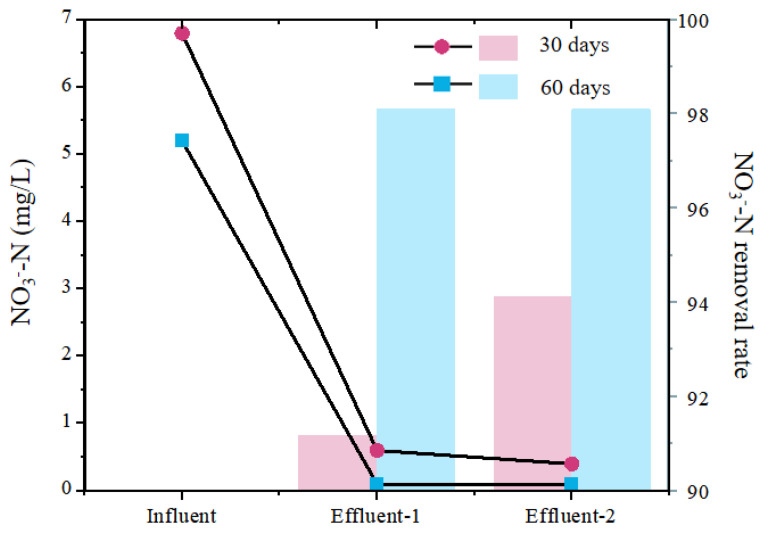
The nitrogen removal performance of the MBfRs with operation times of 30 days and 60 days.

As shown in Figure 2, both parallel MBfRs with the operation time of 30 days and 60 days can obtain good nitrogen removal effects; however, the NO_3_^−^-N removal effect of MBfRs with an operation time of 60 days was significantly better than that of the MBfR with an operation time of 30 days. On the 60th day, the NO_3_^−^-N removal rate reached more than 98.00%, and the NO_3_^−^-N concentrations of influent and effluent were 5.2 and 0.1 mg/L, respectively. On the 30th day, the highest NO_3_^−^-N removal rate was only 94.12%, and the influent and effluent concentrations were 6.8 and 0.4 mg/L, respectively.

This result indicated that the extension of operation time had a certain effect on the functional microorganisms inside the bioreactors. In order to further reveal the reasons for the difference in NO_3_^−^-N removal effects caused by different operation times, the microbial composition and community succession were further analyzed, as outlined in the following sections.

### 3.2. Succession of Relative Abundance 

**Figure 3:**
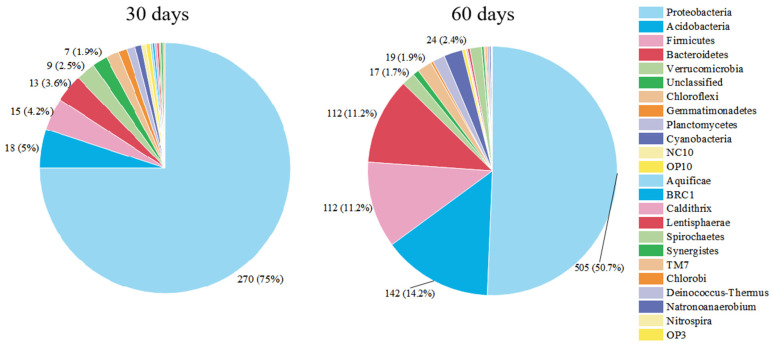
Relative abundance of bacteria (Phylum level) in MBfRs with an operation time of 30 days and 60 days.

The high-density universal 16S rRNA microarray was used to compare the microbial community diversity difference between the MBfRs with 30-day and 60-day operation times. The experimental results revealed a variety of bacterial populations in the MBfRs. The number of OTUs increased significantly in the MBfRs with an operation time of 60 days compared to the MBfRs with an operation time of 30 days, and there were more bacteria at the phylum level. The detected OTU numbers were 360 and 997 for operation times of 30 days and 60 days, respectively. The bacteria in the MBfR at 30 days were separated as 19 phyla, while at 60 days, they were separated as 24 phyla. As shown in Figure 3, *Proteobacteria* was the predominant phylum in both MBfRs. The microbial community composition at the level of the phylum at 60 days was similar to that reported in the related study [18]. *Proteobacteria* are a group of Gram-negative bacteria, and they are the largest and most diverse in the bacterial field. This group includes many bacteria that are closely involved in recycling carbon, nitrogen, and sulfur in the environment [19,20].

When the operation time was prolonged from 30 days to 60 days, the relative abundance of *Proteobacteria* decreased from 75.0% to 50.7%. Meanwhile, with the extension of the operation time, the relative abundance of *Acidobacteria*, *Firmicutes,* and *Bacteroidetes* increased significantly from 5%, 4.2%, and 3.6% to 14.2%, 11.2%, and 11.2%, respectively. Some members of *Acidobacteria* can use D-glucose, D-xylose, and lactose as carbon sources, and *Acidobacteriota* can use both inorganic and organic nitrogen as their nitrogen sources [21]. Bacteria belonging to *Bacteroidetes* can participate in the degradation of a variety of pollutants [22,23]. Some genera of *Firmicutes* are involved in the metabolism of nitrogen and sulfur [24]. The increase in the relative abundance of these bacteria at the phylum level corresponded to the increase in NO_3_^−^-N removal efficiency in the MBfR with an operation time of 60 days.

**Figure 4:**
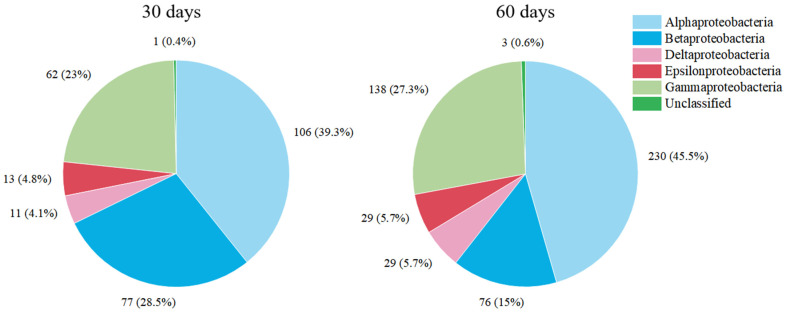
Relative abundance of *Proteobacteria* (class level) in the MBfRs with operation times of 30 days and 60 days.

The microbial composition of *Proteobacteria* at the class level is shown in Figure 4, and the microbial community distribution of *Proteobacteria* was similar in the two MBfRs. This indicated that the community structure of *Proteobacteria* was relatively stable between the two months. *Alphaproteobacteria* was the dominant class in the MBfR, and *Betaproteobacteria* and *Gammaproteobacteria* were also in high proportion. The second and third dominant classes were *Beta-* and *Gammaproteobacteria* with an operation time of 30 days, while the order was reversed with an operation time of 60 days. A relevant study pointed out that *Alpha*- and *Betaproteobacteria* are potential nitrogen fixers, that *Alphaproteobacteria* plays an important role in each step of denitrification, and that *Betaproteobacteria* is very important for ammonia oxidation [25]. However, previous studies had reported the presence of *Gammaproteobacteria* as the predominant microorganism in MBfRs used to reduce nitrates and other contaminants [26,27]. This may be due to differences in the influent composition, operation modes, and operation time.

### 3.3. The Concentration of Microbial Communities

For the analysis of microbial communities in a bioreactor, relative abundance can only indicate the number and proportion of microbial species and does not indicate the concentration of each microbial species, which is important for understanding microbial status in MBfRs. The concentration of bacteria can be accurately reflected using fluorescence intensity, and higher fluorescence means a higher concentration of microbial communities [28]. Microbial concentrations in an MBfR with an operation time of 30 days are shown in Table S1. The bacteria with the top 20 average fluorescence intensity all belong to *Proteobacteria*, and *Betaproteobacteria* is dominant at the class level. However, *Xanthomonadaceae*, with the highest fluorescence intensity, belongs to *Gammaproteobacteria* with a fluorescence intensity of 6743. Many species of the *Xanthomonadaceae* family produce Xanthan gum [29]. Xanthan gum is a kind of polysaccharide that belongs to the component of extracellular polymeric substance; it is closely related to the formation of biofilms in MBfRs [30]. It may be in the growth phase of biofilms at this time.

For the MBfR with an operation time of 60 days, the fluorescence intensity of microorganisms was generally lower than that of the MBfR with an operation time of 30 days (Table S2). This showed that bacterial concentrations were significantly lower on the 60th day than on the 30th day. In addition to *Proteobacteria*, *Firmicutes*, *Acidobacteria,* and *Chloroflexi* are also among the 20 dominant bacteria in MBfRs. *Chloroflexi* has been reported as the backbone-forming agent [31] and it has genes related to denitrification [32]. However, *Proteobacteria* were predominant at the phylum level, and *Alpha*- and *Gammaproteobacteria* were dominant at the class level. Similarly, for the 30-day MBfR, the *Xanthomonadaceae* belonging to *Gammaproteobacteria* had the highest concentration, but the fluorescence intensity was only 3789. This means that biofilm formation was significantly reduced.

The reduction in the dominant bacterial concentration of the MBfR on the 60th day is not in conflict with the improvement of NO_3_^−^-N removal efficiency. It has been reported that by extending the sludge retention time, the sludge yield of the MBR can be significantly reduced, and even zero sludge discharge can be achieved after 300 days of operation. The decrease in sludge production did not negatively affect the processing performance of the reactor [33]. The reason for this phenomenon is that the extension of the operation time leads to the accumulation of microbial metabolites, which inhibits the microbial growth rate and leads to the reduction in biomass. At this stage, the microbes need less energy and oxygen to keep their cells functioning [34]. In this study, the presence of a large number of obligate anaerobes (*Clostridia* of the *Firmicutes* phylum) among the top 20 dominant bacteria in the 60-day MBfR was in line with this point.

Combined with the above analysis of microbial relative abundance and concentration in the MBfRs operated for 30 and 60 days, the similarity in the microbial distribution in the MBfRs on day 30 and day 60 was very low, both in terms of microbial community structure and microbial quantity. Therefore, the extension of operation time leads to a large difference in the distribution of microorganisms in the reactor.

### 3.4. The Phylogenetic Relationships of Microbial Communities

**Figure 5:**
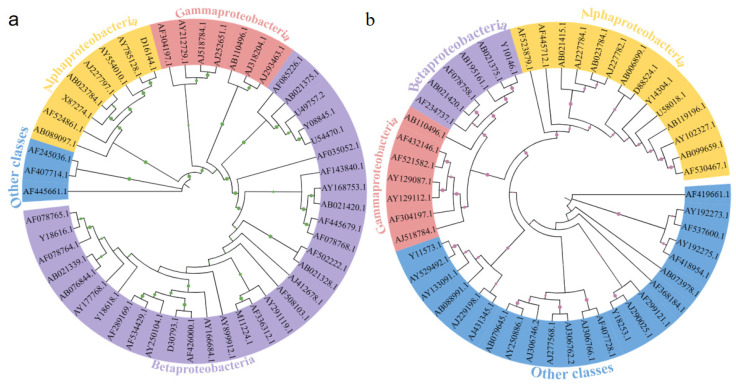
Phylogenetic relationships of 50 dominant universal bacterial sequences retrieved from MBfRs with different operation times: (**a**) 30 days, and (**b**) 60 days.

To further evaluate the succession of microbial community structure in MBfRs with different operation times, a phylogenetic analysis was performed on the identified species. The phylogenetic trees for the neighbor-joining of bacterial species in MBfRs with an operation time of 30 and 60 days were constructed (Figure 5). The sequences of 50 dominant universal bacteria indicated relatively different phylogenetic relationships. The bacteria with Genbank numbers AF445661.1 (*Sphingobacteria*) and AF419661.1 (*Unclassified WS5*) had genetic differences from other members in the two MBfRs. In the MBfR with the operation time of 30 days, the top 50 dominant universal bacteria mainly included *Alpha*-, *Beta-*, and *Gammaproteobacteria*, and only a small number of bacteria from other classes, while in the MBfR with an operation time of 60 days, the number of bacteria from other classes increased significantly.

**Figure 6:**
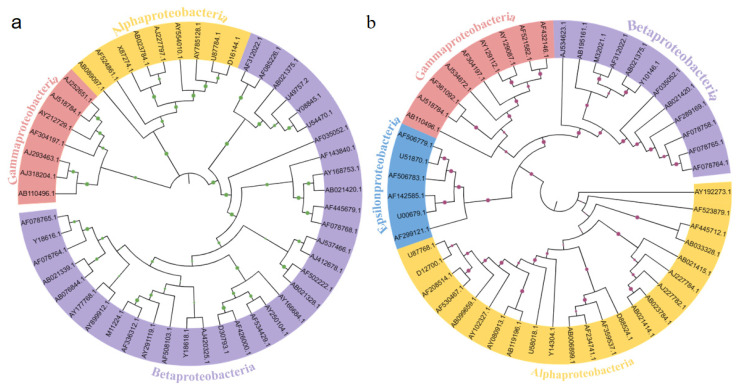
Phylogenetic relationships of 50 dominant *Proteobacteria* sequences retrieved from MBfRs with different operation times: (**a**) 30 days, and (**b**) 60 days.

In order to further evaluate the succession of the *Proteobacteria* microbial community structure with different operation times, the phylogenetic trees of neighbor-joining for *Proteobacteria* were constructed (Figure 6). The sequences of 50 dominant *Proteobacteria* showed significantly different phylogenetic relationships. The bacteria with Genbank number AY192273.1 (*Sphingobacteria*) had genetic differences from other members in the MBfR with an operation time of 60 days. The phylogenetic tree showed that the microbial community in the 30-day MBfR could be divided into three types, *Alpha*-, *Beta*-, and *Gammaproteobacteria*. The microbial community in the 60-day MBfR could be divided into four categories, *Alpha*-, *Beta*-, *Gamma*-, and *Epsilonproteobacteria*. Therefore, it can be seen from the two phylogenetic trees that different operation times will lead to significant differences in the microbial community structure of universal bacteria and the *Proteobacteria* phylum.

### 3.5. The Functional Microbial Communities 

Combined with previous studies on microbial community structure in hydrogen-based MBfRs, the analysis of microorganisms at the genera level can enable us to obtain their corresponding functions. Table 1 shows the functional microorganisms discovered in H_2_-based MBfRs, and their corresponding concentrations are shown.

In H_2_-based MBfRs, hydrogen is the only available electron donor in the system. It is reported that most bacteria belonging to *Comamonadaceae* and *Paracoccaceae* are the typical denitrifying bacteria in these MBfRs, such as *Hydrogenophaga* and *Rhodobacter* [38]. *Hydrogenophaga* uses H_2_ as the electron donor and CO_2_ as the carbon source [39], and some species have denitrification. We detected three species in our sample (*Hydrogenophaga taeniospiralis*, *Hydrogenophaga pseudoflava,* and *Hydrogenophaga electricum*). As described in Section 3.3, the microbial community concentrations in MBfRs at 60 days of operation were generally lower than that at 30 days. However, on day 60, more species of functional microorganisms were detected, such as those belonging to *Comamonadaceae* and *Paracoccaceae*, and had increased from 46 and 24 genera to 73 and 46 genera, respectively. This indicated that with the extension of the operation time, the concentration of microorganisms decreased, but the microbial abundance and diversity further increased, which corresponded to the improvement in the NO_3_^−^-N removal efficiency after 60 days of operation.

## 4. Conclusions

Compared with the MBfR at an operation time of 30 days, the MBfRs at the operation time of 60 days showed significantly higher NO_3_^−^-N removal efficiencies and the nitrogen removal rates reached 98.1%. At the phylum level, *Proteobacteria* was the dominant phylum in both MBfRs at different operation times; however, the composition of the microbial community was quite different. The community composition of *Proteobacteria* at the class level was similar between the two MBfRs. *Alphaproteobacteria* was the dominant class in MBfR, and *Betaproteobacteria* and *Gammaproteobacteria* were also in high proportion.

For the top 20 dominant bacteria with the highest fluorescence intensity, *Proteobacteria* was the dominant group in both MBfRs. *Betaproteobacteria* were the main bacteria at the operation time of 30 days, and many bacteria belonging to the class *Clostridia* appeared in the 60-day MBfRs. According to the analysis of microbial relative abundance and concentration, the similarity in the microbial distribution in MBfRs on day 30 and day 60 was very low. For MBfRs that operated for 30 and 60 days, the phylogenetic relationships of the 50 dominant universal bacteria and *Proteobacteria* were different. With the extension of the operation time, the concentrations of microorganisms decreased, but the microbial abundance and diversity further increased, which corresponded to the improvement in the NO_3_^−^-N removal efficiency at day 60. Through the above analysis, we can conclude that different operation times have significant effects on microbial compositions and community succession.

## Figures and Tables

**Table 1:** Functional microorganisms in MBfRs found in this study.

Genus	Species	Function	Relative Fluorescence Intensity	Sample	Reference
*Marinobacter*	*Marinobacter* *hydrocarbonoclasticus*	Nitrate-reducing bacteria	2112–2098	30d–60d	[27]
*Thiobacillus*	*Thiobacillus denitrificans*	Hydrogenotrophic denitrifying bacteria	1165	30d	[35]
*Hydrogenophaga*	*Hydrogenophaga* *pseudoflava*	Hydrogenotrophic denitrifying bacteria	4766–3036	30d–60d	[35,36]
	*Hydrogenophaga electricum*		4552–2532		
	*Hydrogenophaga* *taeniospiralis*		5552–2864		
*Rhodobacter*	*Paracoccus denitrificans*	Autotrophic denitrifying bacteria	1593–2643	30d–60d	[35,37]

## Data Availability

The data presented in this study are available upon request from the corresponding author.

## Supplementary Materials

The following supporting information can be downloaded at: https://www.mdpi.com/article/10.3390/membranes13030324/s1, Table S1: Average fluorescence intensity of the 20 dominant bacteria in the MBfR (30 days).; Table S2: Average fluorescence intensity of the 20 dominant bacteria in the MBfR (60 days).
