# Supplementary material for: Analysis of Microbial Communities in Membrane Biofilm Reactors Using a High-Density Microarray
Source: Membranes (Basel). 2023 Mar 11;13(3):324. doi: 10.3390/membranes13030324 (PMC10052966; doi:10.3390/membranes13030324)
Supplement: Supplementary file 1 [file membranes-13-00324-s001.zip › membranes-2278679-supplementary.pdf]

Table S1. Average fluorescence intensity of the 20 dominant bacteria in the MBfR (30 days).

class_tax	*order_tax*	*family_tax*	*rep_prokMSAname*	*Average fluorescence intensity*
Gammaproteobacteria	*Xanthomonadales*	*Xanthomonadaceae*	*Dyemonas todaii str. XD10*	*6743*
Betaproteobacteria	*Nitrosomonadales*	*Nitrosomonadaceae*		*5819*
Gammaproteobacteria	*Legionellales*	*Unclassified*	*uranium mining waste pile clone JG37-AG-14 proteobacterium*	*5730*
Betaproteobacteria	*Burkholderiales*	*Comamonadaceae*	*Hydrogenophaga taeniospiralis str. ATCC 49743*	*5552*
Alphaproteobacteria	*Sphingomonadales*	*Sphingomonadaceae*	*Sphingopyxis flavimaris str. SW-151*	*5519*
Betaproteobacteria	*Burkholderiales*	*Comamonadaceae*	*Variovorax paradoxus TG27*	*5437*
Betaproteobacteria	*Burkholderiales*	*Comamonadaceae*	*Comamonas testosteroni str. SMCC B329*	*5419*
Betaproteobacteria	*Burkholderiales*	*Oxalobacteraceae*	*Massilia timonae timone*	*5353*
Betaproteobacteria	*Burkholderiales*	*Comamonadaceae*	*travertine hot spring clone SM1E01*	*5352*
Gammaproteobacteria	*Xanthomonadales*	*Xanthomonadaceae*	*Waste-gas biofilter clone BIyi3*	*5331*
Betaproteobacteria	*Burkholderiales*	*Comamonadaceae*	*Lampropedia hyalina str. DSM 15336*	*5183*
Betaproteobacteria	*Burkholderiales*	*Comamonadaceae*	*Aquaspirillum metamorphum str. DSM 1837*	*5127*
Betaproteobacteria	*Burkholderiales*	*Comamonadaceae*	*Acidovorax facilis str. CCUG 2113*	*5123*
Betaproteobacteria	*Burkholderiales*	*Comamonadaceae*	*napthalene-contaminated sediment clone 76*	*4945*
Betaproteobacteria	*Burkholderiales*	*Comamonadaceae*	*strain isolate str. rJ10*	*4923*
Betaproteobacteria	*Burkholderiales*	*Comamonadaceae*	*hydrocarbon-degrading consortium clone AF1-8*	*4906*
Betaproteobacteria	*Burkholderiales*	*Comamonadaceae*	*Delftia tsuruhatensis str. AD9*	*4855*
Betaproteobacteria	*Burkholderiales*	*Comamonadaceae*	*strain isolate str. rM4*	*4849*
Betaproteobacteria	*Burkholderiales*	*Comamonadaceae*	*Acidovorax defluvii str. BSB411*	*4846*
Alphaproteobacteria	*Rickettsiales*	*Unclassified*	*termite gut homogenate clone Rs-M62 proteobacterium*	*4820*

Table S2. Average fluorescence intensity of the 20 dominant bacteria in the MBfR (60 days).

phylum_tax	class_tax	order_tax	family_tax	rep_prokMSAname	Average fluorescence intensity
Proteobacteria	*Gammaproteobacteria*	*Xanthomonadales*	*Xanthomonadaceae*	*Dyemonas todaii str. XD10*	3789
Firmicutes	*Clostridia*	*Unclassified*	*Unclassified*		3756
Proteobacteria	*Alphaproteobacteria*	*Bradyrhizobiales*	*Beijerinck/Rhodoplan/Methylocyst*	*Beijerinckia indica*	3658
Proteobacteria	*Gammaproteobacteria*	*Legionellales*	*Unclassified*	*uranium mining waste pile clone JG37-AG-14 proteobacterium*	3561
Proteobacteria	*Alphaproteobacteria*	*Rhodobacterales*	*Rhodobacteraceae*	*Rhodobacter sphaeroides str. 2.4.1*	3553
Proteobacteria	*Alphaproteobacteria*	*Rhizobiales*	*Phyllobacteriaceae*		3491
Firmicutes	*Clostridia*	*Clostridiales*	*Peptococc/Acidaminococc*	*anoxic bulk soil flooded rice microcosm clone BSV43 clone*	3481
Acidobacteria	*Acidobacteria*	*Acidobacteriales*	*Acidobacteriaceae*		3464
Proteobacteria	*Gammaproteobacteria*	*Methylococcales*	*Unclassified*	*Methylobacter marinus str. A45*	3443
Firmicutes	*Clostridia*	*Clostridiales*	*Lachnospiraceae*	*termite gut homogenate clone Rs-Q18 bacterium*	3372
Proteobacteria	*Gammaproteobacteria*	*Symbionts*	*Unclassified*	*Seepiophila jonesi symbiont*	3343
Proteobacteria	*Betaproteobacteria*	*Burkholderiales*	*Oxalobacteraceae*	*Paucimonas lemoignei str. ATCC 17989T*	3340
Chloroflexi	*Anaerolineae*	*Unclassified*	*Unclassified*	*DCP-dechlorinating consortium clone SHA-36*	3332
Proteobacteria	*Alphaproteobacteria*	*Bradyrhizobiales*	*Hyphomicrobiaceae*	*Hyphomicrobium aestuarii str. DSM 1564*	3328
Firmicutes	*Clostridia*	*Clostridiales*	*Clostridiaceae*	*Clostridium acetobutylicum str. ATCC 824*	3308
Proteobacteria	*Alphaproteobacteria*	*Caulobacterales*	*Caulobacteraceae*	*Brevundimonas diminuta str. IAM 12691T*	3280
Proteobacteria	*Alphaproteobacteria*	*Bradyrhizobiales*	*Methylobacteriaceae*	*Methylobacterium thiocyanatum str. ALL/SCN-P*	3269
Proteobacteria	*Alphaproteobacteria*	*Rhizobiales*	*Unclassified*		3249
Proteobacteria	*Gammaproteobacteria*	*Unclassified*	*Unclassified*		3245
Firmicutes	*Mollicutes*	*Anaeroplasmatales*	*Erysipelotrichaceae*		3238
